# High blood pressure among adolescents in Africa: A systematic review and meta-analysis protocol

**DOI:** 10.1371/journal.pone.0264728

**Published:** 2022-03-03

**Authors:** Cecilia Amponsem-Boateng, William K. Bosu

**Affiliations:** 1 Department of Epidemiology and Biostatistics, College of Public Health, Zhengzhou University, Zhengzhou, Henan, PR China; 2 Department of Public Health and Research, West African Health Organization, Bobo-Dioulasso, Burkina Faso; University of Mississippi Medical Center, UNITED STATES

## Abstract

**Introduction:**

As high blood pressure (HBP) is often considered an adult disease in Africa, studies on and services for HBP focus on adults to the near-exclusion of adolescents. The dearth of information about the burden of HBP does not favour much attention being paid to it. We, therefore, prepared this protocol to estimate the prevalence and awareness of HBP in adolescents in Africa through a systematic review and meta-analysis.

**Methods:**

We will search several major databases for published and unpublished articles on population-based studies on adolescents living in Africa, aged 10–19 years produced from the year 2000 to date. The included articles will be those that define HBP according to international guidelines using the blood pressure cut-offs of the 95^th^ percentile for age, sex and height or of 130/90 mmHg, depending on the age of the subjects. The study selection as well as the evaluation of the quality of the included articles will be done independently by two reviewers, in line with best practices. We will pool together the prevalence across studies using random effects analysis and assess heterogeneity using meta-regression analysis and sub-group analysis. Sensitivity analysis using a leave-one-out analysis and an evaluation of reporting bias will also be performed. Reporting of our findings will conform to the recommended reporting guidelines.

**Conclusion:**

The findings from our comprehensive systematic review and meta-analysis will provide an up-to-date information on the prevalence of HBP and its awareness among adolescents in Africa and its sub-regions. They could be used to advocate for increased priority to life course approach to the prevention of cardiovascular diseases.

**Systematic review registration:**

PROSPERO CRD42020197946.

## Introduction

Globally, systematic reviews and meta-analysis of studies on hypertension have been undertaken in different age groups. Among children ≤19 years, the global prevalence of high blood pressure (HBP) and prehypertension was 4.0% and 9.7% respectively [1]. Another global meta-analysis estimated a prevalence of 11.2% of HBP in adolescents aged 10–19 years [2]. Systematic reviews and meta-analyses have been undertaken in populous countries among children aged 1–20 years in China [3], those aged 4–19 years in India [4], those aged 3–18 years in Iran [5] and among those aged 10–19 years in Brazil [6] and in India [7].

While systematic reviews and meta-analysis on the prevalence of hypertension in Africa have focused on adults [8–10] and older adults [9, 11–13] and pregnant women [13], to date, we are aware of only one (published in 2017) which has focused on children and adolescents [14]. Systematic reviews and meta-analyses in African countries are, similarly, scarce with one in Nigeria estimating that 5.1% and 8.2% of its children aged 1–19 years have hypertension and prehypertension respectively [15]. This compares with 5.5% and 12.7% respectively among children and adolescents aged 2–19 years in Africa [14]. The dearth of reviews may be because HBP in adolescents is considered less common than in adults. In many African countries, adolescent health concentrates on sexual and reproductive health with little priority to noncommunicable diseases (NCDs). Thus, the World Health Organization recommendation of a life course approach to NCDs is seldom applied [16]. In view of the evidence that childhood hypertension tracks into adulthood, it is important that adequate information is obtained at this early stage to enable appropriate interventions aimed at preventing hypertension in later life [17, 18]. We, therefore, present this protocol to estimate the prevalence and awareness of HBP in the adolescents in Africa as well as its sub-regions and to identify associated risk factors.

Unlike the Noubiap et al. [14] review, our systematic review and meta-analysis will focus on adolescents aged 10–19 years in Africa and will not limit the meta-analysis to only studies which define HBP based on the fourth report of the National High Blood Pressure Education Program Working Group on High Blood Pressure in Children and Adolescents (NHBEPWG) [19] or the American Academy of Pediatrics [20]. Some included studies in the earlier systematic review were not analyzed because their definition of HBP were considered ‘non-acceptable’ [14] with a potential loss of valuable information. Studies in Africa and elsewhere, not only define HBP and pre-hypertension using the 95^th^ and 90^th^ percentile of blood pressure for age, sex and height respectively, but also them using them using the 130/80 mmHg and 120/80 mmHg as thresholds [6, 15]. The latter cut-offs are actually in line with the recommendations of the American Academy of Pediatrics for children aged ≥13 years [20]. The systematic review of hypertension in adolescents in Nigeria included studies in which hypertension was defined as BP> 2 standard deviations of the mean [15].

The review questions to be evaluated will include the following:

- What is the prevalence of HBP in adolescents aged 10 to 19 years and living in Africa?
- What are the risk factors associated with HBP in adolescents in Africa?
- What is the level of awareness of HBP among adolescents in Africa, in those with and without HBP?

## Materials and methods

### Protocol and registration

This protocol is registered at PROSPERO with registration number, CRD42020197946, and will comply with the Preferred Reporting Items for Systematic Reviews and Meta-analyses (PRISMA-P) guidelines (S1 Table) [21].

### Study area

Africa covers 6% of the total earth surface and 20% of the land areas of the earth [22], with nearly 1.3 billion inhabitants as at 2017, representing about 16% of the total global population [23]. The United Nations (UN) estimates that Africa had a population of about 298 million aged 10–19 years representing 22% of the total population in 2019 [24]. The major health problems of adolescents in Africa include HIV, sexual transmitted infections, road injuries, tuberculosis, and diarrhoeal diseases [25].

For statistical convenience, the United Nations Statistics Division categorizes Africa countries into five geographical regions—Eastern, Western, Northern, Southern, and Mid or Central Africa sub-regions, as shown in Table 1.

**Table 1:** United Nations categorization of countries in the African region.

Eastern Africa	Middle Africa	Northern Africa	Western Africa	Southern Africa
British Indian Ocean Territory	Angola	Algeria	Benin	Botswana
Burundi	Cameroon	Egypt	Burkina Faso	Eswatini
Comoros	Central African Republic	Libya	Cabo Verde	Lesotho
Djibouti	Chad	Morocco	Côte d’Ivoire	Namibia
Eritrea	Congo	Sudan	Gambia	South Africa
Ethiopia	Democratic Republic of the Congo	Tunisia	Ghana	
French Southern Territories	Equatorial Guinea	Western Sahara	Guinea	
Kenya	Gabon		Guinea-Bissau	
Madagascar	Sao Tome and Principe		Liberia	
Malawi			Mali	
Mauritius			Mauritania	
Mayotte			Niger	
Mozambique			Nigeria	
Réunion			Saint Helena	
Rwanda			Senegal	
Seychelles			Sierra Leone	
Somalia			Togo	
South Sudan				
Uganda				
United Republic of Tanzania				
Zambia				
Zimbabwe				

Source: United Nations Statistics Division [26]

### Inclusion criteria

Articles will be included if they:

- Cover apparently healthy adolescent population aged between 10–19 years living in Africa
- Are published or unpublished, original articles, abstracts, or conference proceedings which report the prevalence/incidence of HBP among adolescents, defined as blood pressure (BP) ≥95^th^ percentile for age, sex and height for younger children (aged <13 years) or BP ≥130/80 mmHg for older children aged ≥13 years [20]
- Are population-based, cross-sectional, or prospective studies published from January 2000 to present

For multi-country/regional studies, articles will be included if it is possible to extract distinct information on adolescent HBP pertaining to African countries. For studies published in multiple articles, the article with the most relevant and detailed information will be retained.

### Exclusion criteria

Articles will, however, be excluded if they:

- Are case reports, expert opinions, review articles, commentaries, results
- Cover raised non-systemic blood pressure (e.g., pulmonary or portal hypertension)
- Involve adolescents who are obviously ill with acute illness or are living with chronic diseases such as HIV or mental disorders
- Cover adolescents living outside the African region

### Search strategy

Major electronic databases (Medline, Web of Science, CINAHL, Academic Search Ultimate, APA PsycINFO and EMBASE) as well as African Journals Online will be searched for eligible literature. Grey literature will also be searched via Google Scholar. Search terms will be guided by the PICOC approach comprising P for P” for population, “I” for intervention or exposure, “C” for comparator, “O” for outcomes and “C” for context [10, 27]. For the population, the terms will be “children”, “child”, “childhood”, “adolescent”, “paediatric”, “teen”, “teenager”, or “youth”. For the “exposure”, terms will include “prevalence”, “incidence”, “survey”, “risk” or “awareness”. There is no “comparator” term. For the outcome, the search terms will include “hypertension”, “raised blood pressure”, “high blood pressure” or “elevated blood pressure”. For the “context”, the search terms will include “Africa” and the individual names of the African countries (Table 1). The citations yielded from the different search elements will be combined with appropriate Boolean operators “AND” and “OR” (S2 Table).

Where the database permits, age limits will be applied to select the subset of studies pertaining to those aged between 10–19 years. The references of the included articles will be hand-searched to identify further articles of interest.

### Study selection

The study selection will be managed independently by two reviewers using the Covidence software [28], which is designed to remove all duplicate articles imported from the different databases. Titles and abstracts of articles that will emerge from the search strategy will be screened to eliminate ineligible papers (S1 Fig) [21]. All full-text versions of potentially eligible papers will be retrieved and further evaluated to check if they meet the inclusion criteria. The systematic review management software will flag any discordance between the reviewers which will be resolved through mutual consensus.

### Data extraction

Data including the location of study, year of publication, geographical settings, type of study, language, sample size, technique of sampling, participants characteristics, variables, analysis of data and the major findings of studies will be extracted on to a pre-formatted Microsoft Excel. Other data such as socio-demographics, anthropometrics, blood pressure, prevalence and grades of HBP and awareness of hypertension will also be extracted.

Participants with HBP who report having previously been diagnosed by a health professional will be considered to be aware of their HBP [29]. In addition, studies will be screened to determine what proportion of participants understand the meaning of HBP or know a family member who has been diagnosed with hypertension [30]. Where possible, authors will be contacted to provide any critical missing information. Data will be entered by independently and cross-validated by CA-B and WKB.

### Quality assessment

Risk of bias will be evaluated independently by two reviewers (CA-B, WKB) using a tool specifically validated for cross-sectional studies [31]. The tool comprises ten questions which assess internal validity (such as the suitability of case definition, reliability of study instrument, and the application of same measurement methods for all subjects) and external validity issues (such as the representativeness of the sample, participation rate, and the sampling technique). Out of a total of ten points, we will consider a study attaining a total score >8 as having a low risk of bias: score > 8, a score of 6–8 as moderate risk and a score of 0–5 as a high risk of bias: score 0–5 [12, 32]. Inconsistencies in the judgment of reviewers will be mutually resolved by consensus.

### Data synthesis

We will pool the prevalence or awareness of HBP across the included studies using a random-effects model after first stabilizing the variance of the individual studies using the Freeman-Tukey double arcsine transformation in order to minimize the impact of studies with extremely large or small estimates on the pooled prevalence estimate [33]. The heterogeneity between studies will be assessed using the Cochran’s Q chi-squared test statistic [34] and the Higgins and Thompson’s I^2^ statistic [35]. The cut-off I^2^ values of 0%, 25%, 50%, and 75% will respectively represent no, low, moderate, and high level of heterogeneity. The point and 95% confidence interval prevalence estimates of individual studies as well as that of the pooled estimates will be presented in forest plots.

We will perform sub-group analysis and meta-regression analyses to explore the potential sources of heterogeneity. The sub-groups to be analysed will include age group, sex, study design, urban-rural residence and African sub-region. Univariate and multivariate meta-regression analyses will be performed with restricted maximum likelihood estimation and will include study year, publication year, percentage obesity, sample size, definition of HBP and the type of BP device as covariates. Meta-regression analysis requires that there are at least ten studies available for each variable in the model [36].

To assess the robustness of our estimates, we will perform sensitivity analyses in which we will study the effect of omitting one study at a time on the pooled estimate [37]. If there are more than ten included studies, we will evaluate the presence of reporting bias through funnel plot asymmetry and Egger’s test [38]. Funnel plot asymmetry is widely used in meta-analyses of prevalence studies as providing visual evidence of publication bias [39]. The chart is usually a plot of the effect sizes plotted against their standard errors or precisions (the inverse of standard errors). However, it may be inaccurate for assessing publication bias in meta-analyses of proportion studies with low proportion outcomes where plot asymmetry could be mistakenly attributed [40]. In such situations, assessment could be based on funnel plots of study size against log odds or statistical tests such as the Egger or Peter test. Except for the leave-one-out influence analysis to be performed using OpenMeta (analyst) software [41], all statistical analyses will be performed using Stata version 16 [42] with significance level set at 5%.

## Discussion

This protocol seeks to provide an up-to-date estimate of the prevalence of adolescent HBP in Africa. To our knowledge, our study is the first to exclusively estimate the prevalence of HBP in adolescents aged 10–19 years in Africa. While we will include studies with different definitions of HBP, we will estimate a pooled prevalence only among sets of studies which use identical standard definition. Restricting the inclusion criteria to only studies which use a specific definition could lead to potential loss of studies which use acceptable definition of HBP [14]. As a secondary outcome, we will provide an estimate of the level of awareness of hypertension among these adolescents of Africa. We will report the variation in the prevalence by African sub-region (East, West, North, South and Mid/Central). Our search strategy, the choice of databases and the independent screening of studies will ensure that we locate as many studies as are available. We will employ robust methods in the meta-analysis supported by sensitivity analyses. Our analyses will also show regions or countries in which studies on adolescent HBP are scant or lacking.

We anticipate some major study limitations. Significant heterogeneity has been identified in previous meta-analysis of HBP in children and adolescents conducted at the national, regional or global level [1, 14]. In addition, many individual studies tend to measure BP in adolescents at a single visit, and so cannot on the basis of this visit, characterize HBP as hypertension [20]. Nonetheless, HBP and hypertension are frequently used interchangeably in the literature.

Overall, we expect our results on the estimated prevalence and awareness of HBP in the adolescents to draw attention to the cardiovascular health of this population group. In the light of the call for primordial prevention of hypertension in children [43–45], our study will highlight the importance of the life course approach to the prevention and control of hypertension in Africa [46].

## Supporting information

S1 Table Preferred Reporting Items for Systematic Reviews and Meta-Analysis (PRISMA) checklist. (DOCX)

S2 Table Search strategy for Ovid Medline and Ovid Embase databases. (DOCX)

S1 Fig Flow chart for selection of study articles. (TIF)
