# Supplementary material for: High blood pressure among adolescents in Africa: A systematic review and meta-analysis protocol
Source: PLoS One. 2022 Mar 3;17(3):e0264728. doi: 10.1371/journal.pone.0264728 (PMC8893607; doi:10.1371/journal.pone.0264728)
Supplement: S2 Table — (DOCX) [file pone.0264728.s002.docx]

**Supporting Table 2: Search strategy in Ovid PubMed and EMBASE**

| **#** | **Query** |
| --- | --- |
| 1 | exp hypertension/ |
| 2 | (hypertensi* or blood pressure).mp. [mp=ti, ab, hw, tn, ot, dm, mf, dv, kw, fx, dq, nm, kf, ox, px, rx, an, ui, sy] |
| 3 | (cardiovascular or cardiometabolic).mp. [mp=ti, ab, hw, tn, ot, dm, mf, dv, kw, fx, dq, nm, kf, ox, px, rx, an, ui, sy] |
| 4 | (child* or adolescent or school-going or pediatric or youth or teen*).mp. [mp=ti, ab, hw, tn, ot, dm, mf, dv, kw, fx, dq, nm, kf, ox, px, rx, an, ui, sy] |
| 5 | exp Adolescent/ |
| 6 | exp prevalence/ |
| 7 | exp incidence/ |
| 8 | exp risk/ |
| 9 | (prevalence or survey or incidence or cross-sectional or proportion or cohort).mp. [mp=ti, ab, hw, tn, ot, dm, mf, dv, kw, fx, dq, nm, kf, ox, px, rx, an, ui, sy] |
| 10 | (aware* or know*).mp. [mp=ti, ab, hw, tn, ot, dm, mf, dv, kw, fx, dq, nm, kf, ox, px, rx, an, ui, sy] |
| 11 | exp Africa/ |
| 12 | (((Africa or British Indian Ocean Territory or Angola or Algeria or Benin or Botswana or Burundi or Cameroon or Egypt or Burkina Faso or Eswatini or Swaziland or Comoros or Central African Republic or Libya or Cape Verde or Cabo Verde or Lesotho or Djibouti or Chad or Morocco or Ivory Coast or Namibia or Eritrea or Congo or Sudan or Gambia or South Africa or Ethiopia or Democratic Republic of the Congo or Tunisia or Ghana or French Southern Territories or Equatorial Guinea or Western Sahara or Guinea or Kenya or Gabon or Guinea Bissau or Madagascar or Sao Tome) and Principe) or Liberia or Malawi or Mali or Mauritius or Mauritania or Mayotte or Niger or Mozambique or Nigeria or Reunion or Saint Helena or Rwanda or Senegal or Seychelles or Sierra Leone or Somalia or Togo or Uganda or Tanzania or Zambia or Zimbabwe).mp. [mp=ti, ab, hw, tn, ot, dm, mf, dv, kw, fx, dq, nm, kf, ox, px, rx, an, ui, sy] |
| 13 | 1 or 2 or 3 |
| 14 | 4 or 5 |
| 15 | 6 or 7 or 8 or 9 or 10 |
| 16 | 11 or 12 |
| 17 | 13 and 14 and 15 and 16 |
| 18 | remove duplicates from 17 |
| 19 | limit 18 to ("child (6 to 12 years)" or "adolescent (13 to 18 years)" or "young adult (19 to 24 years)") [Limit not valid in Embase; records were retained] |
| 20 | limit 19 to humans |
| 21 | limit 20 to yr="2000 -Current" |
