# Supplementary figures and images for: High blood pressure among adolescents in Africa: A systematic review and meta-analysis protocol
Source: PLoS One. 2022 Mar 3;17(3):e0264728. doi: 10.1371/journal.pone.0264728 (PMC8893607; doi:10.1371/journal.pone.0264728)

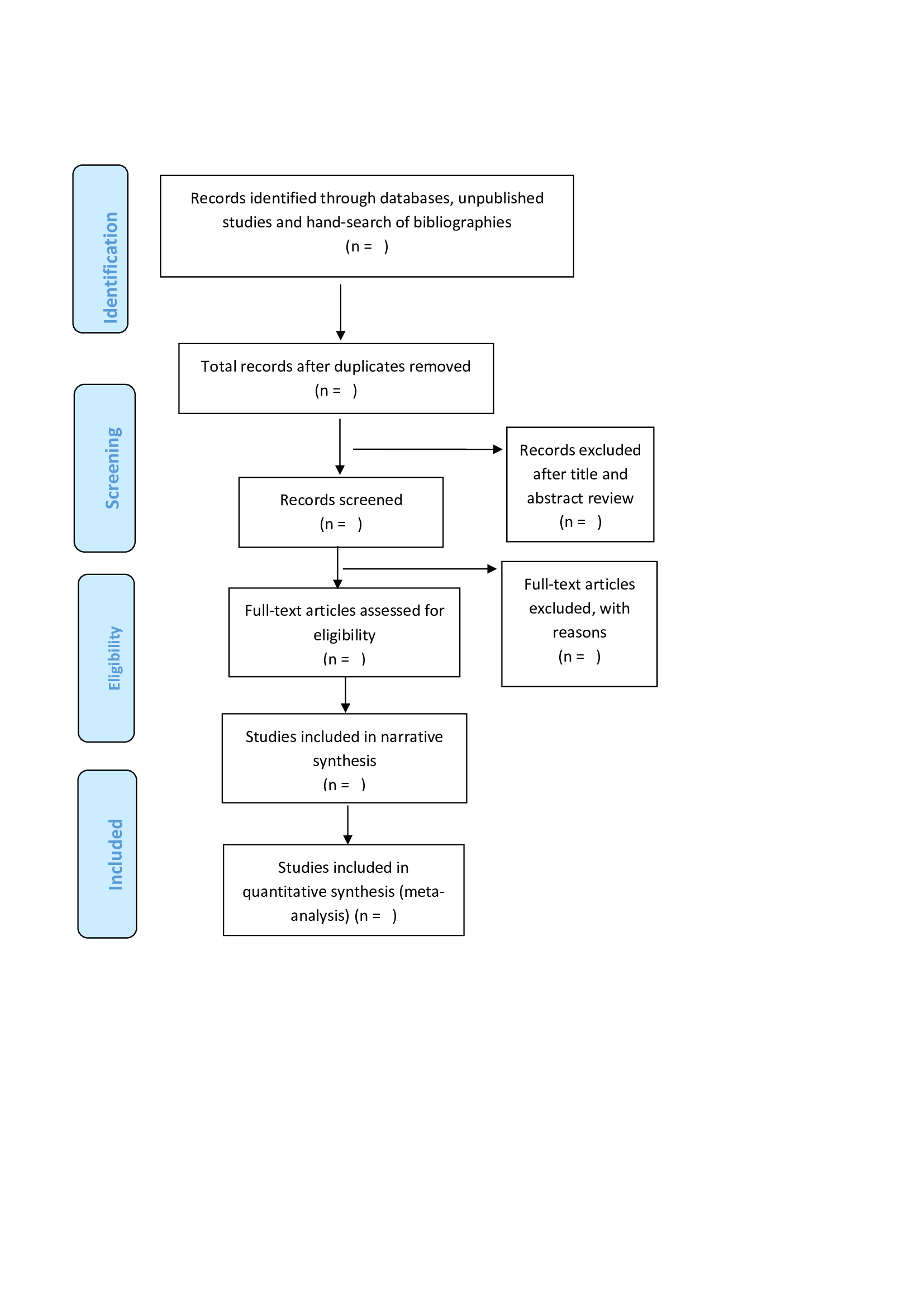

Supplement: S1 Fig — (TIF) [file pone.0264728.s003.tif]
